# ISMB/ECCB 2007: The Premier Conference on Computational Biology

**DOI:** 10.1371/journal.pcbi.0030096

**Published:** 2007-05-25

**Authors:** Thomas Lengauer, B. J. Morrison McKay, Burkhard Rost

**Affiliations:** University of California San Diego, United States of America

## Introduction

The International Society for Computational Biology (ISCB) presents ISMB/ECCB 2007, the *Fifteenth International Conference on Intelligent Systems for Molecular Biology* (ISMB 2007), held jointly with the *Sixth European Conference on Computational Biology* (ECCB 2007) in Vienna, Austria, July 21–25, 2007 (http://www.iscb.org/ismbeccb2007). Now in the final phases of selecting papers, presentations, demonstrations, and posters, the organizers are preparing what will likely be recognized as the premier conference on computational biology in 2007. ISMB/ECCB 2007 has expanded in ways to specifically encourage increased participation from previously underrepresented disciplines of computational biology. This conference will feature the best of the computer and life sciences through a variety of new and core sessions running in multiple parallel tracks, along with an increase in keynote presentations, posters on display throughout the duration of the conference, and an extensive industry exhibition. Special interest group meetings, a satellite meeting, and tutorials all will precede the main conference dates.

## Two Societies Meet

The ISMB conference series was kicked off in 1993 by the vision of David Searls (GlaxoSmithKline), Jude Shavlik (University of Wisconsin Madison), and Larry Hunter (University of Colorado). A few years down the road, ISMB had established itself as a primary event in computational biology and triggered the founding of ISCB, the International Society for Computational Biology (http://www.iscb.org). ISCB has been organizing the ISMB conference series since 1998. While ISCB evolved into the only society representing computational biology globally, its flagship conference has become the largest annual forum focused on computational biology worldwide (Table 1). The ECCB conference series was conceived by T. Lengauer (MPI for Informatics, Saarbrücken), H.-P. Lenhof (Saarland University), and M. Vingron (MPI for Molecular Genetics, Berlin) in 2002 and since then organized annually by a panel of European computational biologists (http://bioinf.mpi-sb.mpg.de/conferences/eccb/eccb.htm). ECCB is the only pan-European conference series in this field. At Glasgow in 2004, ISMB and ECCB pioneered a common meeting chaired by Janet Thornton (EBI Cambridge) and David Gilbert (University of Glasgow) that continues to be perceived as one of the most successful meetings ever by many standards. As a result of the first union in 2004, the two societies are now committed to joining together whenever ISMB will be held in Europe, to further the fellowship and mutual benefit achieved through a combined organizational effort. One positive effect on the two societies has been the strong bond evolving through the common event.

**Table 1:**
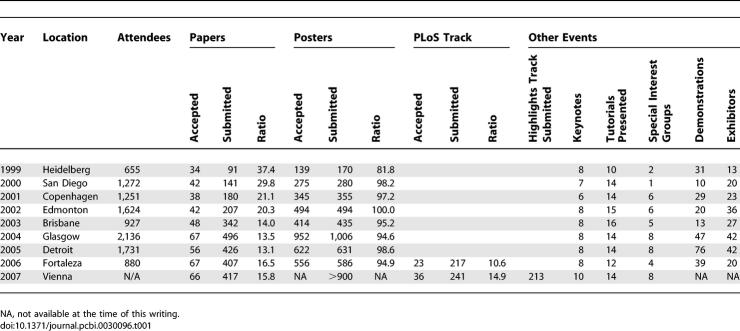
ISMB History by the Numbers

## Two Cultures Intersect

Furthering the success of that initial union, ISMB/ECCB 2007 has expanded in ways to specifically encourage increased participation from previously underrepresented disciplines of computational biology. The major challenge for this interdisciplinary field is that two cultures with very different ways of publishing intersect at computational biology meetings such as ISMB/ECCB: computational scientists publish their most important results in rigorously reviewed proceedings of meetings; the lower the ratio between accepted/submitted, the more valued the publication. In many cases, publication in proceedings of conferences on computer science are valued more highly than those in peer-reviewed scientific journals. In contrast, life scientists publish their best work in peer-reviewed journals with the highest possible impact; journals with higher impact are ranked higher and publications are not coupled to presentations at any meeting. Open access publications attach the value to the individual publication rather than to the unspecific forum of this publication (bad papers can be published in highly ranked proceedings and journals). Thereby, these open access publications may ultimately offer a way out of this clash of publication cultures. However, this solution does not suffice to address the needs of all members of a Society rooted firmly in both cultures.

A multitrack solution to attract both communities has continued to develop since its first implementation at ISMB 2002 in Edmonton, Canada, chaired by David Wishart (University of Alberta). Further expansion today offers even greater opportunities for scientific programming of the conference to include new and relevant areas of research. These goals have guided the organization of the 2007 meeting in many new ways.

## New: Special Sessions

The new *Special Sessions Track* (http://www.iscb.org/ismbeccb2007/program/specialsessions) enables in-depth focus on emerging and important areas. Special Sessions will cover a wide spectrum of topics: genomic database ethics, cheminformatics, genetic networks, computational epigenetics, RNA, and methods in systems biology. For example, the session on *Computational Approaches to the Modern RNA World* chaired by Ivo Hofacker (University of Vienna) will provide a full day focused on the state of the art in RNA bioinformatics to highlight many of the still-open problems that present opportunities for future research. With noncoding RNAs moving from the status of a molecule of minor consequence to the rank of high importance for understanding regulatory function, demand for RNA-related bioinformatics tools continues to rise. This session, as all Special Sessions, will review the current state of a specialized field, and will explore growing opportunities for computational biologists and needs for new tools and methodologies.

## New: Highlights Track

A newly conceived *Highlights Track* (http://www.iscb.org/ismbeccb2007/highlights) is of work that has recently been published and that has either already impacted biology or is likely to do so. Work from highly ranked journals and from journals traditionally more frequented by experimental biology is welcome. As are the other two Viennese novelties, namely the Special Sessions Track and the Industry Track (below), the *Highlights Track* is a pilot project aimed at increasing diversity and adding quality. While it will supposedly require more than one ISMB meeting to conclude whether or not these pilot projects succeeded, the opportunity to present recently published work has already created quite a response from the traditional ISMB attendee, the community leaning more toward the biology side of this interdisciplinary field, and from the panel of experts selecting the talks for presentation. As of this writing, the selection process is under way, and this new track promises presentations based on papers directly impacting work in the field with relevance for molecular and medical biology. Papers from high-impact journals such as *Science, Nature,* and the official ISCB journal, *PLoS Computational Biology,* have been submitted. In all, more than 60 journals were cited among the submissions received, ranging from *Bioinformatics* to *PNAS,* from *Cancer Research* and the *Journal of Critical Care* to *Physical Review Letters* and *Theoretical Biology and Medical Modelling*.

## New: Industry Track

The third Viennese novelty is the *Industry Track* (http://www.iscb.org/ismbeccb2007/industry), for talks more relevant and interesting for colleagues from industry. This track induces a new forum for the meeting of academia and industry in a venue that highlights innovative applications and practical impact studies of Life Science Informatics; it is chaired by Reinhard Schneider (EMBL Heidelberg). Each selected talk is intended to describe a scientific problem from a business perspective, including the approach used to address the problem, the current state of the project, an evaluation of the benefits, and plans for future developments. These presentations are tailored to give attendees the opportunity to view scientific approaches through an industrial lens, which may prove especially valuable to a young researchers' understanding of how Life Science Informatics is used in the business sector.

## Expansion of Keynotes

An increase and refocus of keynote presentations provided a forum for opening ISMB more toward biology at ISMB 1999 in Heidelberg, chaired by T. Lengauer (MPI for Informatics, Saarbrücken). At Vienna, the number of *Keynote Presentations* (http://www.iscb.org/ismbeccb2007/keynotes) has again been increased to provide additional insights into open areas of research from experimental perspectives. Two presentations feature the annual ISCB award winners: Eran Segal (Weizmann Institute) and Temple Smith (Boston University); eight others sample outstanding findings in contemporary biology: Søren Brunak (Technical University of Denmark), Stephen K. Burley (SGX Pharmaceuticals), Michael Eisen (University of California Berkeley), Anne-Claude Gavin (EMBL Heidelberg), John Mattick (University of Queensland), Erin O'Shea (Howard Hughes Medical Institute Harvard University), Renée Schroeder (University of Vienna), and Terry Speed (University of California Berkeley). These lectures will review hot news in areas such as statistics, structural biology and structural genomics, protein interactions, function prediction, RNA, gene expression, mass spectrometry, and transcriptional network structure and function.

## High-Quality Papers

Traditionally, the major event at the ISMB meetings has been the presentation of original papers published in the ISMB proceedings. Initially published by the Association for the Advancement of Artificial Intelligence Press (AAAI Press), the chair of ISMB 2001 in Copenhagen, Søren Brunak, pioneered the publication of the proceedings in the journal *Bioinformatics* as a means of increasing the value of such publications for the more biology-oriented members of the field. As is common practice for computer science, all material published in the proceedings is novel, and publication in the proceedings precludes the publication of this material in any other peer-reviewed journal or meeting. A record number of 417 original manuscripts were submitted to ISMB/ECCB 2007. In a rigorous review process, modeled on the editorial review in scientific journals, 66 (16%) original research *Papers* (http://www.iscb.org/ismbeccb2007/program/papers) were selected for presentation in Vienna. Papers will be presented in two parallel tracks. The paper-reviewing process put increased weight on work that opens new directions and is likely to impact molecular and medical biology in general. All papers selected for oral presentation will be published in the conference proceedings as part of a regular online issue of *Bioinformatics* under the open-access model, i.e., will be made freely available.

## PLoS Track

An initiative from Phil Bourne (University of California San Diego) contributed toward making ISMB 2005 in Detroit, chaired by David States and Brian Athey (both of the University of Michigan), a success by the addition of a *PLoS Computational Biology Late Breaking Poster Session*. At ISMB 2006 in Fortaleza, Brazil, chaired by Goran Neshich (Embrapa/CNPTIA), the concept was slightly altered to become *PLoS Track of Oral Abstracts,* which attracted 217 original submissions of novel work; 24 of those were presented at the meeting. Highly rated at ISMB 2006, the *PLoS Track* (http://www.iscb.org/ismbeccb2007/plostrack) will again be an integral component of ISMB/ECCB. It will feature oral presentations based on submitted abstracts that are scientifically novel and appeal to a broad audience of computational biologists and biologists. As of this writing, a group of area chairs drawn from the *PLoS Computational Biology* editorial board, supported by additional invited area chairs and reviewers, is in the process of selecting the final slate of talks. Barbara Bryant (Millennium Pharmaceuticals) chairs this event, as she did successfully in 2006. Presentations will primarily represent as-yet-unpublished works that demonstrate exceptional scientific merit with a focus on the application of computational methods leading to important biological conclusions. As such, this does not preclude publishing the work elsewhere, thus appealing to the computer science and experimental biology communities.

## Demonstrations

*Demonstrations* (http://www.iscb.org/ismbeccb2007/demonstrations) have also become an integral part of the ISMB and ECCB conference series, and are now also offered at other bioinformatics conferences. Demos allow academic institutions as well as for-profit organizations to showcase their software and/or hardware in a hands-on format to audiences of up to 50 participants. The Demo sessions have proven to be in high demand, and have consistently added a valuable aspect of the conference for both presenters and attendees. At Vienna, Shoba Ranganathan (Macqauarie University) will chair the presentation of demonstrations.

## Posters Full-Time

For the first time in many years, all *Posters* (http://www.iscb.org/ismbeccb2007/posters) accepted for presentation at ISMB will be on exhibit during the entire length of the meeting. Over the years, ISMB had grown to a size that has made it impossible to display all accepted posters during the entire conference. Yet, there is no disagreement on the enormous value all posters bring to the conference. For the first time in several years, we are in a facility with the capacity to accommodate several hundred posters on display at the same time. Therefore, we are especially pleased to ease the viewing of posters by allowing all attendees the opportunity to view and absorb the abundance of research being presented in a single venue. Multiple author sessions will round out the opportunity to further explore posters of specific interest to any attendee. Marco Punta (Columbia University) will chair the selection of posters in Vienna.

## Parallel Scheduling

Except for the plenary talks by keynote presenters, all tracks will run in parallel, allowing attendees the ability to probe deeper into their specific areas of interest and come away with fresh ideas and the possibility of newfound collaborators. While this concept was first introduced at ISMB 2002, Vienna brings a new degree of parallelism by running at least seven parallel sessions: two for original *Papers,* one for *PLoS Track,* one for *Special Sessions,* two for *Highlights Tracks,* and one for *Industry Track* and *Demos*.

## Before and around the Main Meeting

ISMB/ECCB 2007 is also preceded on July 19–20 by seven special interest group meetings (*SIGs*) (http://www.iscb.org/ismbeccb2007/program/sigs), in parallel with a *Satellite Meeting* (http://www.iscb.org/ismbeccb2007/program/sigs/#3dsig) taking place July 19–20. Hershel Safer (Weizmann Institute) chairs this event. An integral pillar of ISMB from the beginning was the day reserved for the presentation of *Tutorials* (http://www.iscb.org/ismbeccb2007/program/tutorials), chaired by Janet Kelso (MPI for Evolutionary Anthropology, Leipzig). The tutorials at Vienna will run in parallel with the 3rd ISCB Student Council Symposium (*SCS3*) on July 21; Manuel Corpas chairs this event (University of Manchester). Each of these preconference meetings offers additional opportunities to learn and network with a specific group of peers of similar interests and goals.

Attendees traveling with family members and/or children will find this year's event offers a variety of optional tours and activities during the meeting, and extended tours on dates surrounding the conference. Specifically for those traveling with children, we are working on providing childcare options and information that would enable parents to attend the meeting while children are safely entertained.

## Registration

ISMB/ECCB 2007 is expected to draw ~2,000 attendees to Vienna to take part in the world's largest and most scientifically comprehensive bioinformatics meeting of the year. ISCB members gain significant *ISMB/ECCB 2007 Registration* (http://www.iscb.org/ismbeccb2007/registration) discounts, yet nonmembers are offered a complimentary, one-year membership as part of their higher nonmember fees. The early bird registration discount period ends June 1 for all registration categories, although registration will remain open through the conference dates. We hope to see you there. 

## Abbreviations

ECCB: European Conference for Computational Biology
ISCB: International Society for Computational Biology
ISMB: Intelligent Systems for Molecular Biology

